# On the Computing Potential of Intracellular Vesicles

**DOI:** 10.1371/journal.pone.0139617

**Published:** 2015-10-02

**Authors:** Richard Mayne, Andrew Adamatzky

**Affiliations:** Unconventional Computing Group, Faculty of the Environment and Technology, University of the West of England, Bristol, United Kingdom; University of Campinas, BRAZIL

## Abstract

Collision-based computing (CBC) is a form of unconventional computing in which travelling localisations represent data and conditional routing of signals determines the output state; collisions between localisations represent logical operations. We investigated patterns of Ca^2+^-containing vesicle distribution within a live organism, slime mould *Physarum polycephalum*, with confocal microscopy and observed them colliding regularly. Vesicles travel down cytoskeletal ‘circuitry’ and their collisions may result in reflection, fusion or annihilation. We demonstrate through experimental observations that naturally-occurring vesicle dynamics may be characterised as a computationally-universal set of Boolean logical operations and present a ‘vesicle modification’ of the archetypal CBC ‘billiard ball model’ of computation. We proceed to discuss the viability of intracellular vesicles as an unconventional computing substrate in which we delineate practical considerations for reliable vesicle ‘programming’ in both *in vivo* and *in vitro* vesicle computing architectures and present optimised designs for both single logical gates and combinatorial logic circuits based on cytoskeletal network conformations. The results presented here demonstrate the first characterisation of intracelluar phenomena as collision-based computing and hence the viability of biological substrates for computing.

## Introduction

Collision-based computing (CBC) is a form of unconventional computing in which travelling localisations represent data—the presence of which in a specific location represents a logical ‘1’ (true) and *vice versa*—which are conditionally routed to represent an output state. When two objects collide, it can be said that computation has been achieved as signal routing is altered.

CBC is best demonstrated with Fredkin and Toffoli’s CBC billiard-ball model (BBM) [1], in which hypothetical billiard balls of equal mass and dimensions that travel along the grid lines of a Cartesian lattice at uniform speed may collide with each other, altering their final trajectories and hence the output of the billiard ball machine. Designed to exploit the laws of physics in order to maximise computational efficiency, the BBM is a reversible (time-invertible), conservative computing paradigm.

A computing device comprised of billiard balls will of course never be a viable alternative to a conventional computer, but rather research in the field will inspire future electrical computer designs and drive the development of unconventional computing substrates whose range of uses will extend beyond those of extant architectures. Indeed, in addition to billiard balls, a great many theoretical and experimental collision-based computing systems have been presented using such diverse media as pliable soft spheres [2], cellular automata [3, 4] and even live soldier crabs [5].

Inspired by Margolus’ soft sphere model (SSM) modification of the BBM [2], which differs from the BBM in that spheres compress on impact and travel as one entity for a finite amount of time, we designed the following investigation into the viability of live cells as a substrate for implementing CBC or CBC derivative paradigms. The plasmodium of slime mould *Physarum polycephalum* was utilised as the research organism.

**Fig 1:**
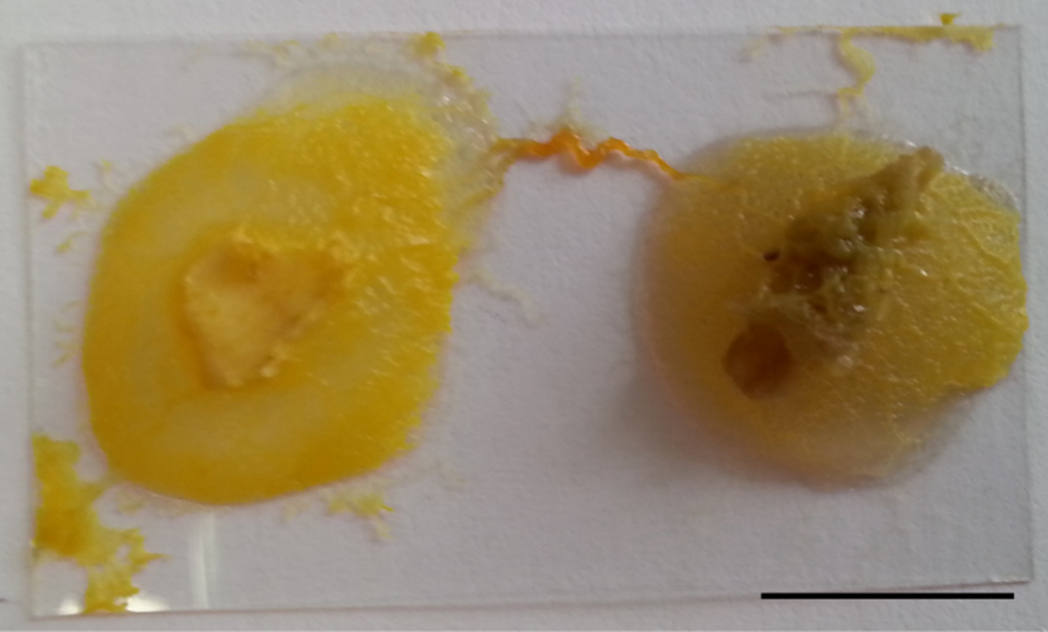
Photograph of the *P. polycephalum* plasmodium (yellow material) cultivating two ‘islands’ of agar substrate overlying a glass coverslip. Note how the organism forms a tube-like structure linking the two agar blobs, which is discoloured following microinjection with the fluorescent calcium indicator, Fura-2. Scale bar = 10mm.

*P. polycephalum* is a true (acellular) slime mould that exists as a macroscopic multinucleate ameoba-like eukaryotic organism when in its plasmodial (vegetative) life cycle phase (Fig 1). Slime mould is much-lauded in its value as an unconventional computing substrate [6], but our choice in utilising is was simply by virtue of it being a giant eukaryotic cell whose cultivation is rapid, frugal, safe and bereft of ethical considerations.

Intracellular computing is a contentious topic: although organisms can be said to function in a manner analogous to computers (e.g. solving problems of arithmetic or logic), the way in which they undertake computation is so divergent from the *in silico* equivalent that any direct comparison between the two is, at best, unhelpful. Unconventional computation is dependent on creative interpretation of the natural world: in this investigation, we observe and interpret intracellular phenomena in the language of computation—regardless of whether the organism can be said to undertake conventional computation.

Intraplasmodial calcium stores were regarded as cellular ‘information’ due to our previous observations and historical literature indicating that slime mould contains significant amounts of calcium which is transported through the organism in vesicles [7, 8]. Calcium is a well known secondary messenger with defined roles in many life processes, see [9–11].

From a computing perspective, any chemical that provokes a response within an organism can be thought of as carrying ‘information’ as it provides a means of coupling an operation to an effect, or more broadly, environment to entity. Furthermore, as the delivery, release and response to such a substance are quantifiable phenomena, output recognition is aided. We are by no means the first to recognise that calcium can be viewed as a component of an excitable chemical processor [12, 13], although other authors have only presented theoretical models based on the concept of reaction-diffusion computing.

## Materials and Methods

Stock cultures of *P. polycephalum* (strain HU554 × HU560) plasmodia were cultivated on 2% non-nutrient agar (NNA) plates at 22 ± 2°C in the absence of light. Plasmodial tubes were prepared by creating two 1mL ‘islands’ of 2% NNA on a large glass coverslip with a gap of c. 10mm separating them. A 20mm^2^ sample of plasmodium, taken from its anterior margin, was removed with a scalpel blade and placed onto one agar blob. The coverslip was then placed in a 9cm plastic Petri dish, which was sealed with paraffin film and left in the dark for 48 hours to propagate to the second agar island, forming a tube between the two (Fig 1).

The fluorescent calcium dyes, Fura-2 and Calcium Green-5N (Life Technologies, USA), were prepared in distilled water at concentrations of 5mM and 1mM respectively, and were introduced into the *P. polycephalum* plasmodium via microinjection using hollow glass needles with a tip diameter of c. 30*μ*m and a CellTram microinjection system (Eppendorf, Germany). Approximately 750nL of dye solution was delivered. Samples were imaged immediately after microinjection.

Confocal imaging was performed with a Zeiss Axiovert 200 inverted microscope combined with a Perkin Elmer Ultraview ERS FRET-H spinning disk confocal microscopy system. Details of image post-processing are listed in the appendices.

## Results

**Fig 2:**
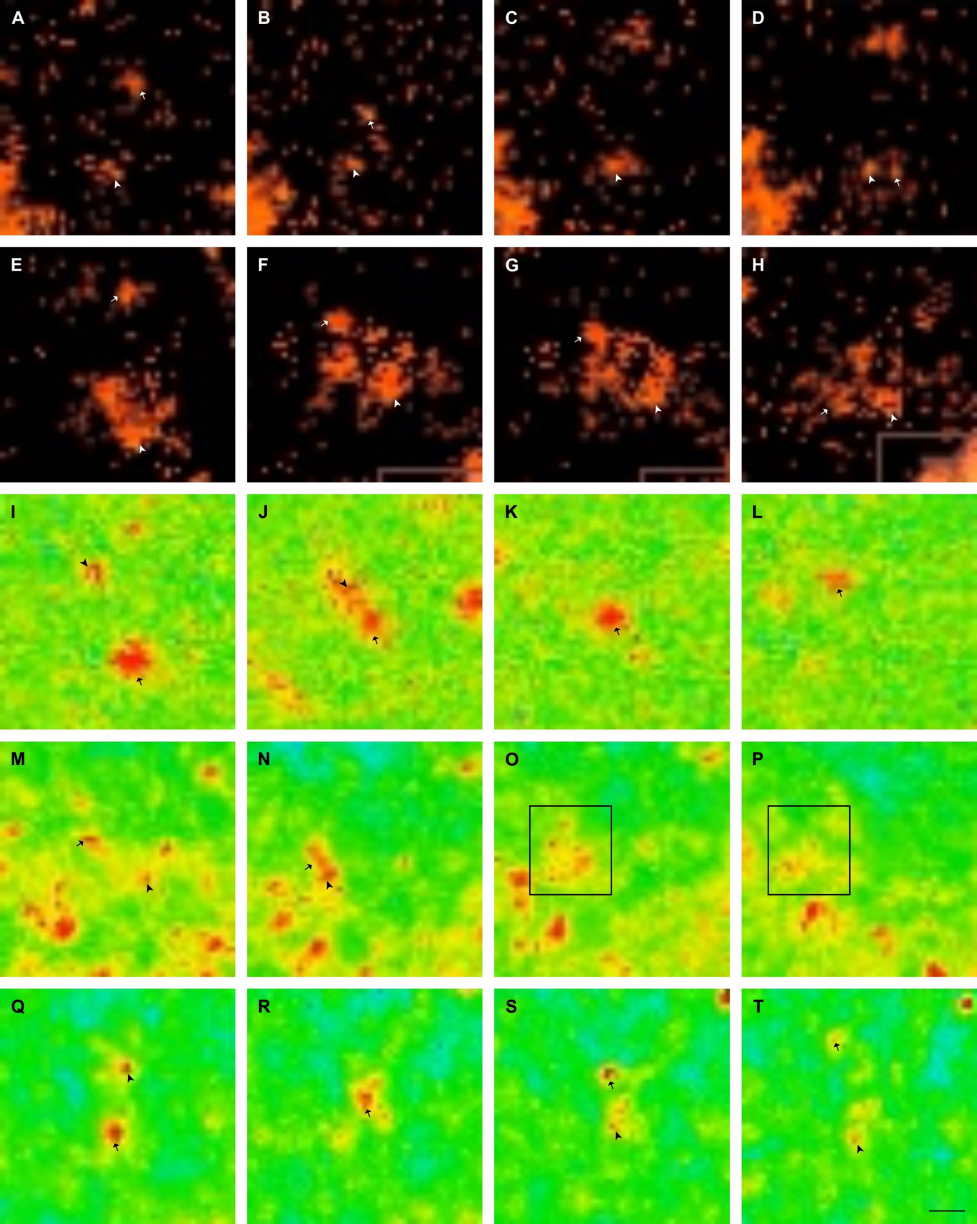
Snapshots of confocal video footage showing calcium vesicle collisions. (A–D) TI. The vesicles collide and reflect. (E–H) TIIa. Note the rotation of the fused object. (I–L) TIIb. The arrowhead-marked vesicle appears to merge into the arrowed vesicle. Upon colliding, the incident vesicle temporarily reverses the direction of its movement against the flow of cytoplasm. (L–P) TIII. Vesicle contents dissipate into the cytoplasm in the fourth pane. (Q–T) TIV. Both vesicles continue to move as if they did not actually collide. (A–H) Fura-2, (I–T) Calcium Green-5N. Scale bar = 10*μ*m, time steps approx. 250ms.

Calcium was found to travel through the plasmodial cytoplasm in discrete, spherical deposits and tended to travel in pre-determined directions, as opposed to taking random routes through the cytoplasm, at a rate of approximately 5*μ*m s^−1^. Collisions of these quantities of calcium were observed as regular occurrences. The types of collision observed may be summarised as follows, based on the observation of 42 collisions (Fig 2):

1. **Type I: Reflection**—57.1% of all collisions; two vesicles collide and ricochet. The incident paths are divergent from their apparent initial course.
2. **Type IIa: Fusion, adhesion**—9.5%; following collision, both vesicles appear to cling to each other but their appearance is that of two separate structures. The vesicles may dissociate after an indeterminate length of time.
3. **Type IIb: Fusion, assimilation**—14.3%; as with type IIa, but the contents of one are noticeably assimilated into the other. The shape of the resulting vesicle is still spherical but must appear to be approximately the same volume of its two constituent vesicles.
4. **Type III: Unloading (annihilation)**—9.5%; following a type I or type IIb collision event, the contents of the vesicle disperse immediately following the collision.
5. **Type IV: Unknown**—9.5%; no apparent outcome from collision, or collision did not occur despite appearance, e.g. if vesicles passed in close proximity.

Collisions whose outcome was unclear due to the vesicles passing out of the microscope’s depth of field were discounted. Localisations of calcium substantially larger than surrounding counterparts were also discounted as no measures for distinguishing between the endoplasmic reticulum and vesicles were utilised.

## Discussion

### Identification and characterisation of vesicles

The spherical quantities of calcium were deduced to be vesicle-bound due to their comparative size, elastic interactions with other similar objects, indications from literature and their propensity to travel down distinct paths through the cytoplasm. These well-circumscribed pathways are highly likely to be the cell’s cytoskeletal network, as vesicle transport is mediated by the cytoskeleton in other protists [14] as well as plant and mammalian cells [15, 16]. It should be noted, however, that the majority of eukaryotic cells do not contain such an abundance of calcium-filled vesicles, the role of which in slime moulds is analogous to that of a striated muscle cell’s sarcoplasmic reticulum [17]. The cytoskeleton was not tagged with fluorescent proteins in this study so as not to induce deleterious effects to the health of the organism and/or disrupt vesicle transport mechanisms, although we refer the reader to Ref. [18] which details a study into plasmodial cytoskeleton topology and functionality as an intracellular data network.

Both tubulin microtubules and actin microfilaments transport vesicles through a cell via motor proteins—dyneins and kinesins in the case of microtubules and myosin for microfilaments [16, 19]—bound to the surface of the vesicle that physically ‘walk’ or ‘slide’ the structure along the cytokeletal protein chain. Thus we may state that collisions occur on a three-dimensional planar surface in Euclidean space, the cytoskeleton, analogous to the manner in which objects travel along grid lines in the BBM and SSM.

### Computing within the vesicle collision model

Let us describe how computing may be implemented with vesicles on cytoskeletal highways with a hypothetical collision event under the assumption that a system for adequately controlling vesicle collisions were developed. Consider an abstracted cytoskeletal protein chain. It has multiple branches that articulate onto it (which may or not be composed of the same protein) via intermediate link proteins: vesicles may travel onto or exit our original cytoskleletal protein via these branches depending on the orientation of the branch, the branch protein type and/or the type of motor proteins residing on the surface of the vesicle (we direct the reader to the following review of vesicle targeting [20]). Collisions may occur when two vesicles travel on the same protein in opposing directions or the same direction but at different speeds, or when two vesicles meet following the convergence of two paths via a branch.

The collision outcome is determined by some as-of-yet unelucidated factor/s but could include the quantity of calcium in each vesicle relative to their critical capacities, vesicle-surface proteins, the variety of cytoskeletal protein the vesicles travel down, the total velocity of collision and/or the angle of collision. To address a salient criticism of the work presented here, we do not differentiate between actin, tubulin or intermediate filament-related collisions, or indeed any collisions that may occur independently of the cytoskeleton (see following sections), and hence we present our preliminary model statistically based on relative probabilities of collision type.

The predominant reflection-type (Type I) collisions may result in either one or both vesicles assuming a new trajectory. Vesicles appear to deform and temporarily merge upon collision for approximately 50–100 ms (e.g. Fig 2A–2D), indicating that the resulting reflection occurs due to elastic recoil. The change in incident vesicle trajectory is likely to be caused by their diversion onto a different branch of the cytoskeletal network, which may result from their changing ‘track’ on the original protein chain (e.g. the motor protein’s feet ‘skip’ to a different protein molecule). This is consistent with our knowledge of vesicle targeting as vesicle movement is generally towards the cell’s periphery (see following section): the cytoskeleton is extremely dense, meaning that it is likely to be highly redundant and hence multiple paths are compatible with a vesicle’s targeting mechanisms. As such, TI collisions may be used to simulate collision events according to Margolus’ SSM [2], subject to a few alterations in what we will name the ‘vesicle collision model’ (VCM).

Type II (fusion) and III (annihilation) collisions cannot be directly equated to conservative logical functions as the quantity of vesicles changes following a collision. Both mechanisms may still hold practical use, however e.g. as delay elements, 2–to–1 fan-in or stop operations. Furthermore, the natural incidence of vesicle annihilation would suggest that it is a route towards controlled calcium release which, if adequately controlled, could hold singular value in provoking a measurable output by the cell.

Type IV collisions likely arise from two vesicles passing very close to each other on the same protein chain or on adjacent chains, although it is difficult to ascertain the exact mechanism with any certainty. On the assumption that vesicles pass close to each other on the same path, we can equate this with the SSM mechanism of two signals crossing at points between vertices that do not result in a collision: this is an attractive mechanism for signal synchronisation in practical VCM circuits.

### Experimental characterisation of collisions as Boolean logic

It must be made emphatically clear that cells do not themselves compute with vesicles, but we conceive that collision phenomena may be characterised as *in vivo* Boolean operations.

**Fig 3:**
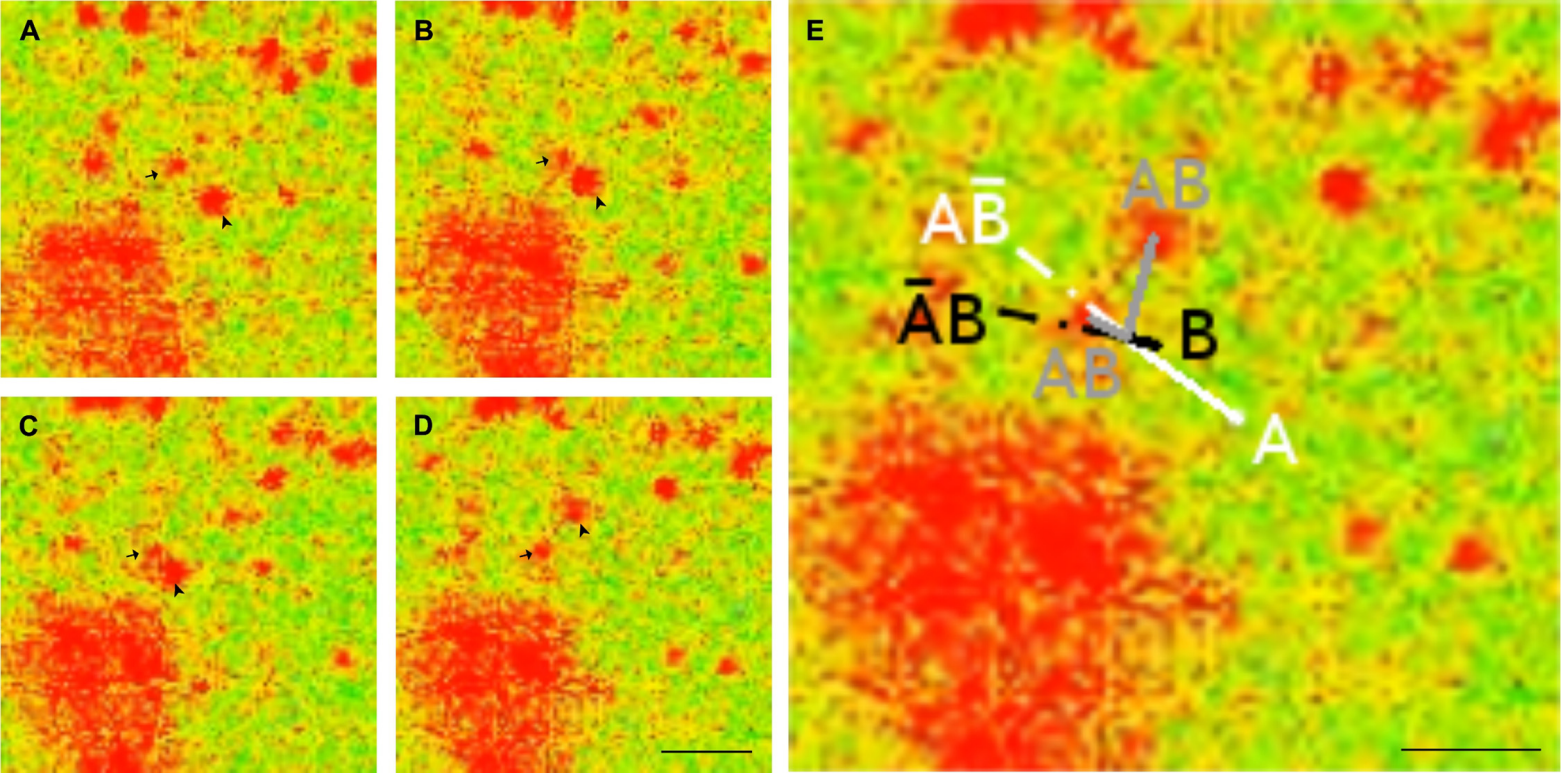
Confocal micrographs to illustrate how vesicle trajectories may be interpreted as an implementation of the interaction gate following a TI collision. (A–D) TI collision event. (E) Enlarged frame from image [D] with trajectories of vesicles shown, where grey components represent the output of the collision shown (i.e. both inputs = 1) and dashed lines show the presumed unperturbed course for each vesicle. Calcium Green-5N staining, scale bars [A–D], [E] = 10*μ*m, time steps approx. 250ms.

It is, for example, apparent that TI collisions can be characterised as a realisation of Fredkin and Toffoli’s interaction gate. This gate may be configured to function as the and gate when its output is recognised as true as a result of the input configuration ⟨*A*∧*B*⟩ (Fig 3), although it may also be considered as a variety of other gates depending on how the output is interpreted. Conversely, a TIII collision’s output is consistent with that of an xor gate—i.e. $\left\langle A\wedge \bar{B}\rangle \vee \langle \bar{A}\wedge B\right\rangle$, due to the annihilation of both signals when the input is configured as ⟨*A*∧*B*⟩. These examples illustrate that slime mould vesicle interactions can be characterised as a computationally-universal set (i.e. and and xor) of Boolean operations.

### Practical implementation of the VCM

It is now pertinent to discuss how these natural mechanisms may be hijacked for practical use and the challenges to be overcome towards this goal. As with other CBC models, the topology of the interaction environment will dictate the types of logical operation that can be implemented therein. Minute manipulation of cytoskeletal protein chains has been achieved [21], but only *in vitro*. Furthermore, whilst cytoskeletal growth is essentially programmable—through the use of of actin binding proteins that drive tip growth of the actin network during pseudopodic extension [22], although controlled polymerisation technology is still very much in its infancy [23]—the assembly of designer cytoskeletal circuitry is likely to be difficult to achieve in practice.

This highlights the need to design VCM circuitry using logical gates based on common topological features so that all of the necessary components are already present within a model cell. It may also be the case that the point at which the vesicles collide within the circuitry does not need to be exact due to the expected redundancy of cytoskeletal networks: as long as a collision occurs within a given section of cytoskeletal protein chain, there are several incident paths along which a vesicle may be reflected that lead to the same point.

With regards to achieving vesicle synchronisation, as slime mould calcium transport is heavily linked to shuttle streaming [24]—the rhythmic bi-directional flow of cytoplasm instigated by muscle protein contraction and hence intracellular calcium flux—manipulation of streaming feedback mechanisms will be the most feasible approach to this end. Indeed, if the streaming mechanism is considered to be an abstracted biochemical oscillator whose frequency can be altered with relative ease [25, 26], then clocked circuit designs would seem to be the simplest route to achieving synchronisation. That said, synthetic approaches such as *in vitro* synthesis of vesicles and their associated membrane proteins may also be profitable to explore [27, 28]. The introduction of delay elements would greatly aid the problem of synchronisation.

Although vesicle transport is a cooperative effort between microtubules and microfilaments, it is crucial to emphasise that the properties of the circuit—principally, its topology—will be entirely dependent on the protein/s from which they are formed. The following designs for practical circuits are entirely based on actin microfilaments arranged in well-characterised conformations. Actin was chosen as over tubulin as we have previously emphasised its role in intracellular computation [18]: whilst the high strength of actin networks [29, 30] and their recent demonstration to participate in purposeful long-range vesicle transport [31] as well as local transport make actin an apparently exemplary VCM medium, its choice over tubulin or indeed mixed-protein networks is essentially arbitrary. Crucially, our devices are based on the assumption that they are integrated into dense, highly interconnected, stable cortical networks, wherein directional vesicle transport occurs.

**Fig 4:**
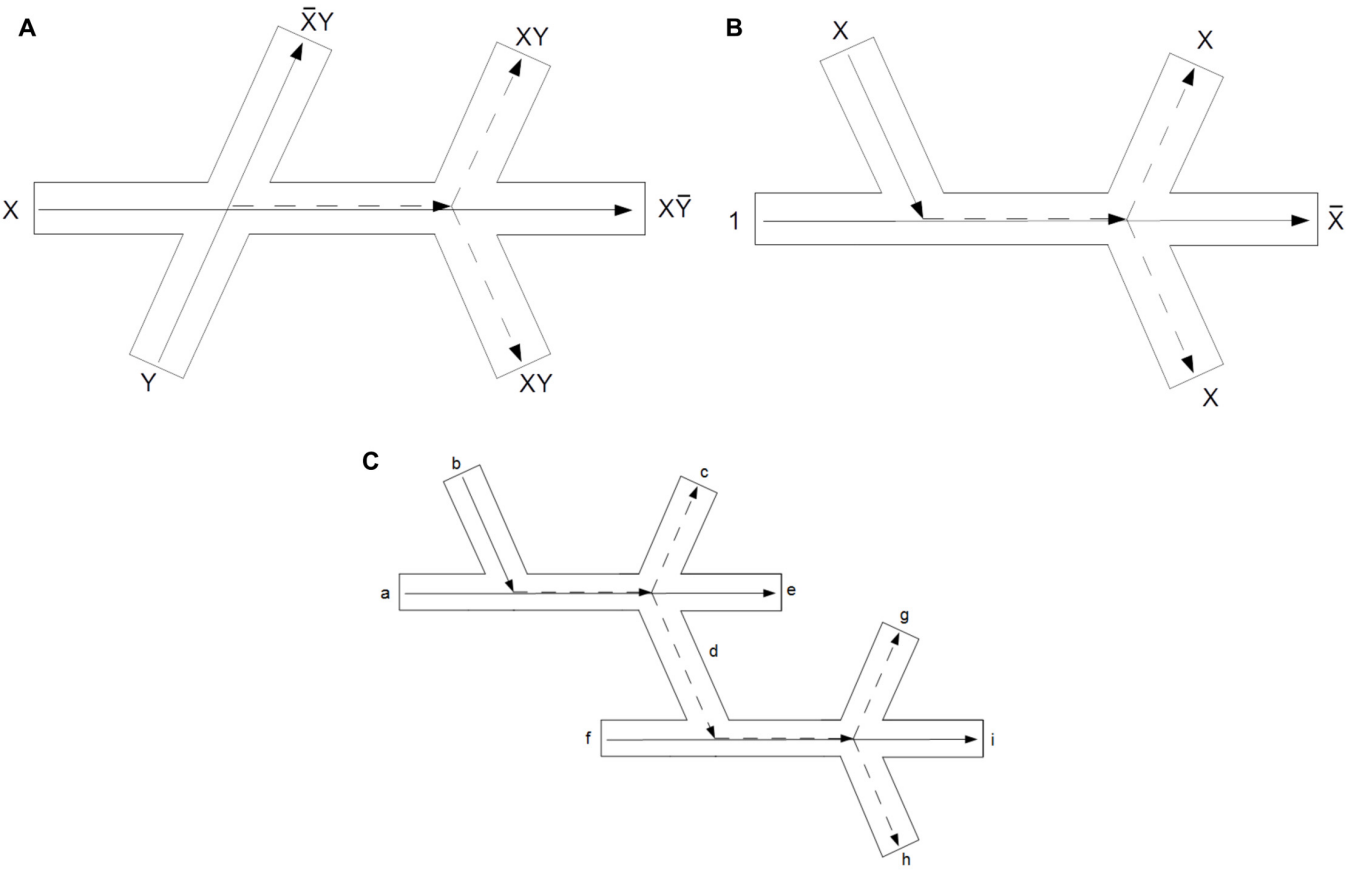
Schematic diagrams of three vesicle collision circuits based on TI collisions occurring on actin structures. Solid arrows represent starting trajectories which continue to their final destination if no other signal is present and dashed arrows represent the trajectories resulting from a collision. (a) The interaction gate functioning as an and gate, formed from one cross-shaped junction and two branches. (b) A not gate formed from three branches. Note that a constant ‘control signal’, denoted by the number ‘1’ is required. (c) A fan-out gate formed from two not gates. Again, control signals are used, ‘a’ and ‘f’, but the output configuration is altered to allow for signal duplication, with ‘c’, ‘g’ and ‘h’ representing outputs, ‘e’ and ‘h’ are garbage bits.

**Fig 5:**
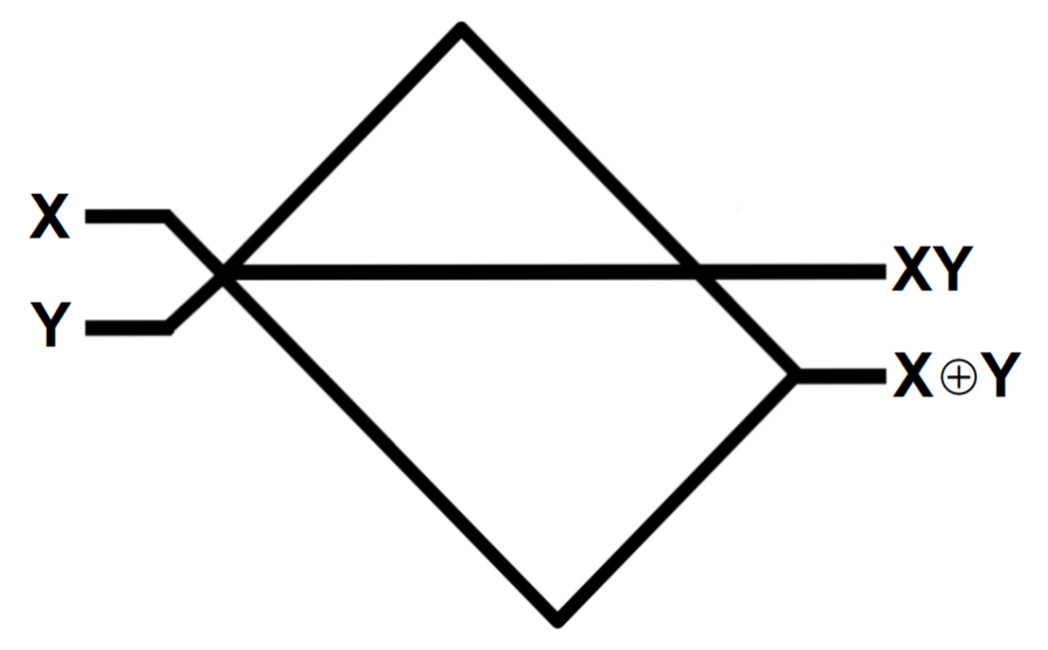
Schematic diagram for a half-adder circuit where TIIb (fusion; assimilation) collisions are utilised. If only one vesicle is present, it will pass down the xor (Sum) pathway: note that in this instance, the fact that it crosses the and (Carry) pathway does not matter as it will not meet and collide with another vesicle. If two vesicles are present, they meet and fuse, leading to one signal being present on the Carry path. Path lengths not to scale.

By way of demonstration, Fig 4 contains schematic diagrams for actin-based interaction and, not and fan-out logical circuits which demonstrate that with suitable control over cytoskeletal topology (or indeed a probabilistic approach based on common protein conformations) a computationally universal set of gates could be implemented. These designs capitalise upon TI collisions occurring at ‘X-shaped’ junctions, which can be realised by microfilament cross-linking proteins such as spectrin or filamin, and branches created by Arp2/3 complex. There are an enormous range of possibilities when designing ‘useful’ cytoskeletal circuits (indeed, only two-dimensional schemes are listed here for ease of recognition) and in acknowledgement of this, the designs presented detail a feasible schematic for a collision type we have experimentally observed (the interaction gate) and two we have not. The not and fan-out make use of constantly true control signals, i.e. a regular, synchronous supply of vesicles, which despite not having been observed to occur naturally would be a plausible method for overcoming the notorious difficulties in implementing logical disjunction and fan-in/out in conservative logic [32]. Furthermore, if a certain degree of control over the type of collision is exerted (e.g. through selective expression of vesicle surface proteins), VCM circuit design becomes significantly more flexible, as demonstrated in Fig 5, which shows a half adder circuit based on the principle of TIIb collisions.

In his treatise on reversible computing, Toffoli [33] emphasised that the concept of function composition must equate to 1-to-1 mapping of inputs in physical models of computing, meaning that the use of the fan-out function in our devices invalidates this criterion: in other words, the devices presented here that utilise signal replication are not isentropic systems. Despite classical models of CBC being hypothetically non-dissipative, this is not the case with the VBM as, being essentially powered by chemical energy (mostly the hydrolysis of ATP), a certain amount of heat will be lost from the system (see following subsection).

For completeness, we estimate an experimental model of an adder circuit using nand (formed from not plus and) and fan-out gates to be *c.* 1*μ*m in length (at approximately 10 gates long, each consisting of two branching sections spaced a realistic distance of c. 50nm apart [34, 35]) with an operation time ranging between 0.1–1.0s, using maximum and minimum measured estimates of vesicle transport velocity ranging across all known types of cytoskeletal vesicle transport [16, 36].

It should be noted that vesicle direction—both in the correct general direction and along the correct microfilament in orthogonal junctions—is dictated by the central microfimalent’s orientation towards the cell surface, as the vast majority of vesicle transport in actin networks is directed by the myosin motor proteins that link vesicles to the network [31]. As previously mentioned, vesicle targeting and possibly also collision type is dictated by the physical properties of the vesicle, principally the proteins bound to its surface. We conceive that through the selection of desirable vesicle properties (whether this is through extraction from live (possibly genetically-altered) cells [37, 38], differential centrifugation of cell homogenate [39, 40] or *in vitro synthesis*) [27], when coupled with careful introduction of vesicles into specific areas of the system—likely through microinjection for *in vivo* systems or specialised, synchronous input lines for *in vitro systems*—is a plausible method for achieving complete control over a VCM system whose interaction environment topology is suitably prepared.

### On the robustness of biological computing substrates

It is an inescapable fact that a living system will contain an extremely large number of degrees of freedom. This implies that elucidating the precise interactions between each individual component is virtually impossible—especially in a system such as this where several underlying principles of operation are incompletely characterised. On face value, therefore, it would be reasonable to state that slime mould intracellular vesicle collisions are nondeterministic and hence the system is of limited use as a computing substrate. In the original description of the BBM [1], however, it was noted that in ‘real world’ systems, the degrees of freedom may be divided into a small number of highly regular (mechanical) modes—those that obey strict physical laws—and a much larger number of disordered (thermal) modes: the laws which the former obey are necessarily well-defined (rooted in classical mechanics as opposed to statistical laws) and hence predictable (and also technically time-invertible, although this is a moot point when discussed in relation to a system that does not attempt to implement conservative logic). Thus, when we observe non-reversible, non-conservative behaviour, this is the result of from energy transfer from mechanical to thermal modes (damping).

We conceive, therefore, that vesicle dynamics are programmable on the basis that their behaviour is predominantly controlled by its deterministic modes, which are likely to include vesicle-surface proteins as previously discussed. *In vivo*, initial conditions of the system are such that the energy levels of the mechanical modes are vastly greater than those of the thermal, as energy must be able to flow preferentially from the former to the latter. This energy gradient is maintained by constant signal regeneration: this is unsurprising as biological organisms tend to be extremely good at maintaining homeostatic equilibrium, but critically this implies that a hijacked cell or an *in vitro* system’s behaviour will be inherently predictable if the same conditions are maintained.

### Summary

The motivation for research into unconventional/biological computing includes, briefly, our attempts to curtail the rapid approach to the physical limitations of the materials in silicon-based architectures, the apparent computing power to energy consumption ratio of biological substrates, the polluting nature of conventional computer manufacture and the emergent properties of biological substrates, such as self-assembly/organisation, massive parallelism and the huge potential information density of macromolecules, the retrieval of which poses significantly fewer issues concerning heat dissipation. Devices that utilise whole or components of live organisms are not putative successors or even competitors with general purpose computers, but will provoke nature-inspired designs for artificial systems and find niche uses in a range of research disciplines such as biomedical science and sensing.

We have demonstrated here the viability of a living biological system for implementing a computationally universal collision-based computing system using vesicles filled with signalling molecules. Vesicle collisions may be used by slime mould as a component of important intracellular signalling processes, but we suggest that this natural mechanism may also be ‘hijacked’ for implementing Boolean logic. Future work will consist of differential observation of collisions in separate actin and tubulin networks, network analysis of collision circuits (e.g. with directed network graphs derived from vesicle motion tracking [41]), vesicle synthesis and tagging, *in vitro* growth of cytoskeletal networks and investigations using different cell types.

## Appendix

### Image post-processing

Photographs were captured with an Olympus SP-820UZ digital camera. All micrographs were post-processed with Volocity (Improvision, USA) and underwent colour assignment and contrast enhancement. Deconvolution was not used. Image plates were produced with Cytosketch (Cytocode, NZ). Unprocessed image files will be made available on request.
